# Supplementary material for: Lung SORT LNPs enable precise homology-directed repair mediated CRISPR/Cas genome correction in cystic fibrosis models
Source: Nat Commun. 2023 Nov 11;14:7322. doi: 10.1038/s41467-023-42948-2 (PMC10640563; doi:10.1038/s41467-023-42948-2)
Supplement: Supplementary file 1 — Supplementary Information [file 41467_2023_42948_MOESM1_ESM.pdf]

Supplementary Information

Lung SORT LNPs Enable Precise Homology-Directed Repair Mediated CRISPR/Cas Genome Correction in Cystic Fibrosis Models

Tuo Wei, Yehui Sun, Qiang Cheng, Sumanta Chatterjee, Zachary Traylor, Lindsay T. Johnson, Melissa L. Coquelin, Jialu Wang, Michael J. Torres, Xizhen Lian, Xu Wang, Yufen Xiao, Craig A. Hodges, and Daniel J. Siegwart.*

Supplementary Figures

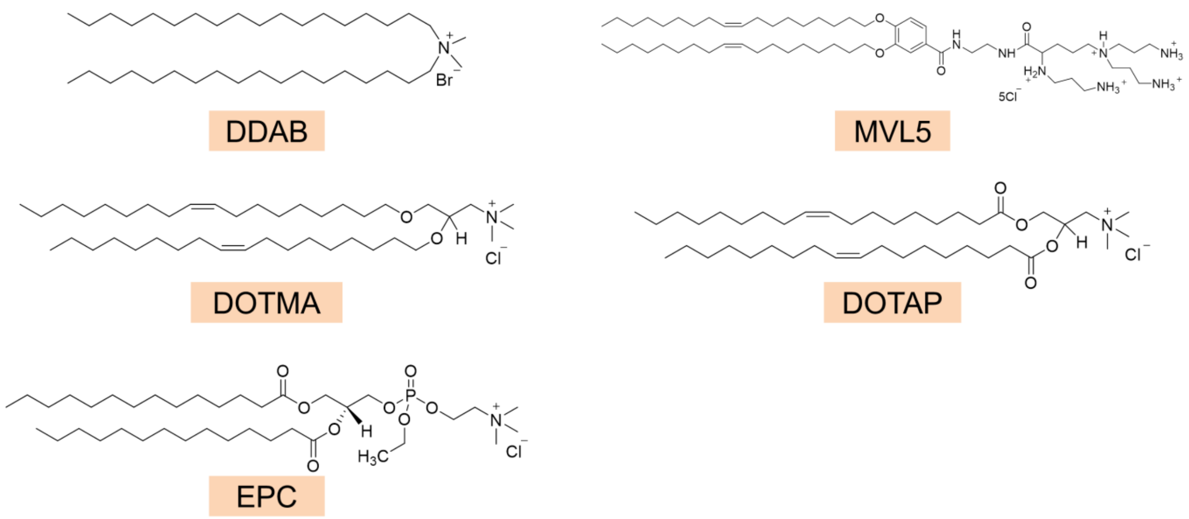

Supplementary Fig. 1 | Chemical structures of permanently cationic lipids used, including DDAB, DOTMA, EPC, MVL5, and DOTAP.

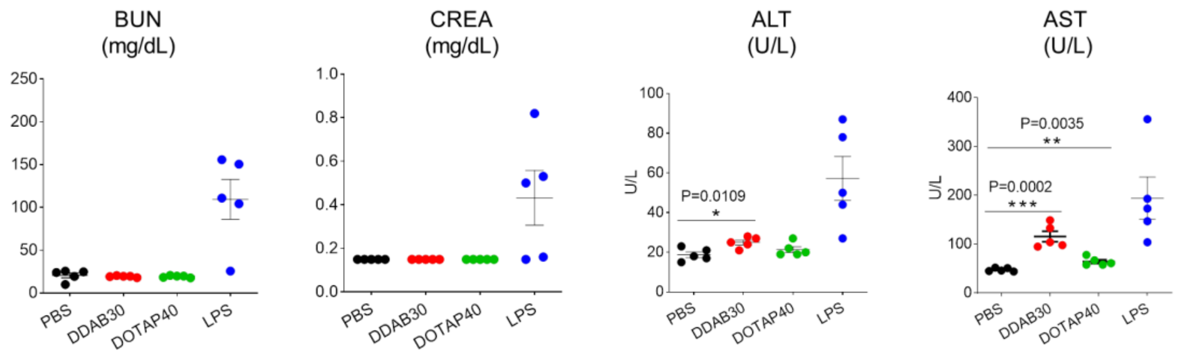

Supplementary Fig. 2 | DOTAP40 showed lower toxicity than DDAB30 *in vivo*. *In vivo* toxicity of DOTAP40 and DDAB30 LNPs were evaluated by measuring kidney function parameters (BUN and CREA) and liver function parameters (ALT and AST) in mouse serum after 24h of treatment (1.5mg kg^{-1} mCherry mRNA, i.v., 40/1 (total lipid/total RNA)). PBS treatment (i.v.) was used as a negative control, and lipopolysaccharide (LPS, i.p., 5mg kg^{-1}) treatment was used as a positive control. Please note that Fig. 2g has been reproduced above in Supplementary Fig. 2 to assemble relevant data together for enhanced clarity. Data are presented as mean ± s.e.m. (n= 5 biologically independent animals). A two-tailed unpaired t-test was used to determine the significance of the comparisons of data (*P< 0.05; **P< 0.01; ***P< 0.001).

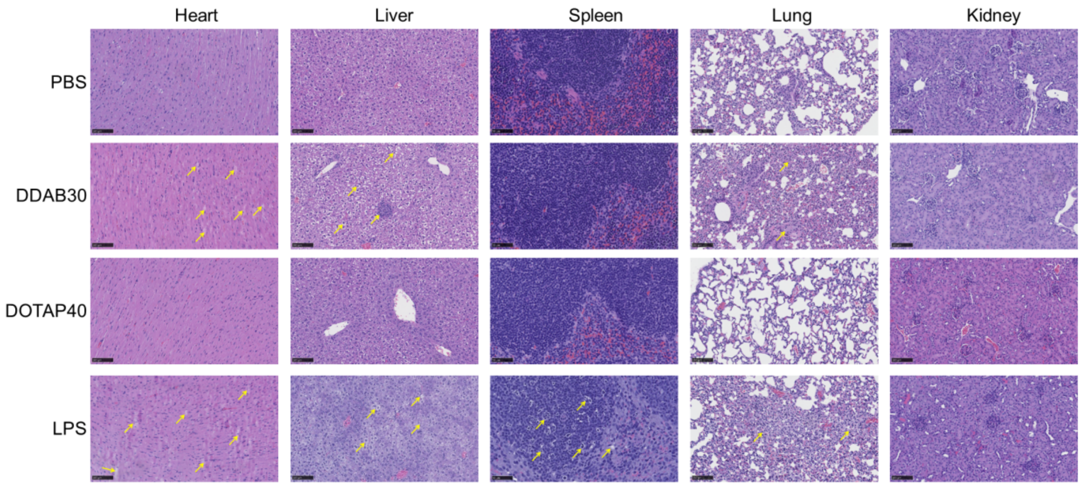

Supplementary Fig. 3 | Representative H&E staining images of mouse tissues after treatment with PBS, DDAB30, DOTAP40 and LPS. Mice were treated with DOTAP40 and DDAB30 LNPs (1.5mg kg^{-1} mCherry mRNA, i.v., 40/1 (total lipid/total RNA)). PBS treatment (i.v.) was used as a negative control, and lipopolysaccharide (LPS, i.p., 5mg kg-1) treatment was used as a positive control. LPS group mice were sacrificed at day 2 after treatment and other groups were killed at day 5. At sacrifice time point, all the organs (heart, liver, spleen, lung and kidney) were collected and sectioned for H&E staining. Obvious tissue damages in heart, liver and lungs were observed when treated with DDAB30 LNPs and LPS, while DOTAP40 LNPs treatment showed low toxicity. Scale bar: 100 μm.

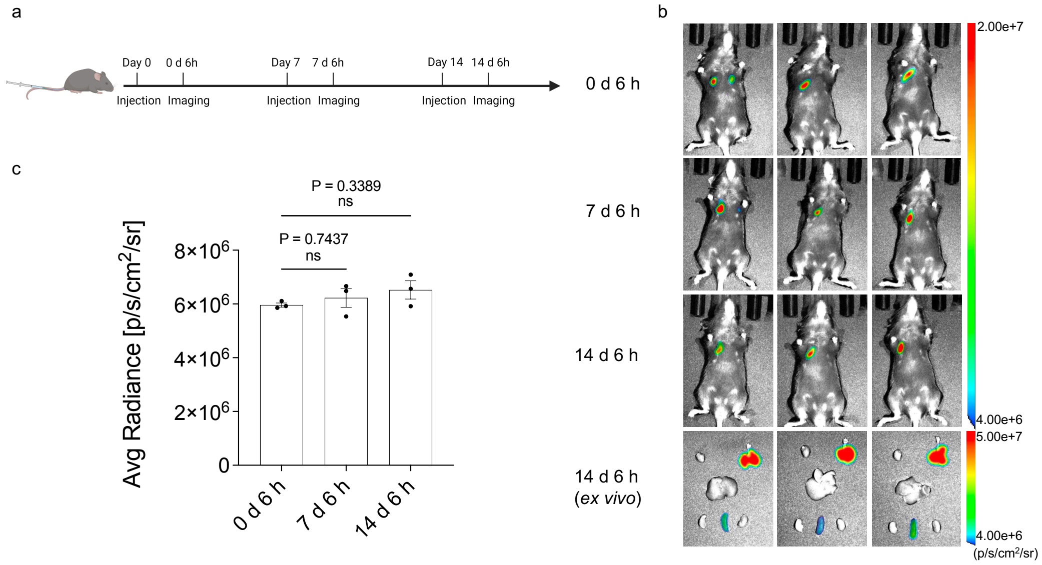

Supplementary Fig. 4 | DOTAP40 LNPs allowed repeat dosing without loss of efficacy. a, C57BL/6 mice were treated with DOTAP40-Luc (Luc mRNA, 0.1 mg kg^{-1}, i.v., 20/1 (total lipid/total RNA)) three times in total, one week apart. Whole body *in vivo* imaging (b) and quantification of luciferase expression (c) was performed 6 hours after each injection. *Ex vivo* major organ imaging (b) was performed 6 hours after the third injection. Data are shown as

mean±s.e.m. (n=3 biologically independent animals). Two-tailed unpaired t-tests were used to determine the significance of the comparisons of data (*P< 0.05; **P< 0.01; ***P< 0.001).

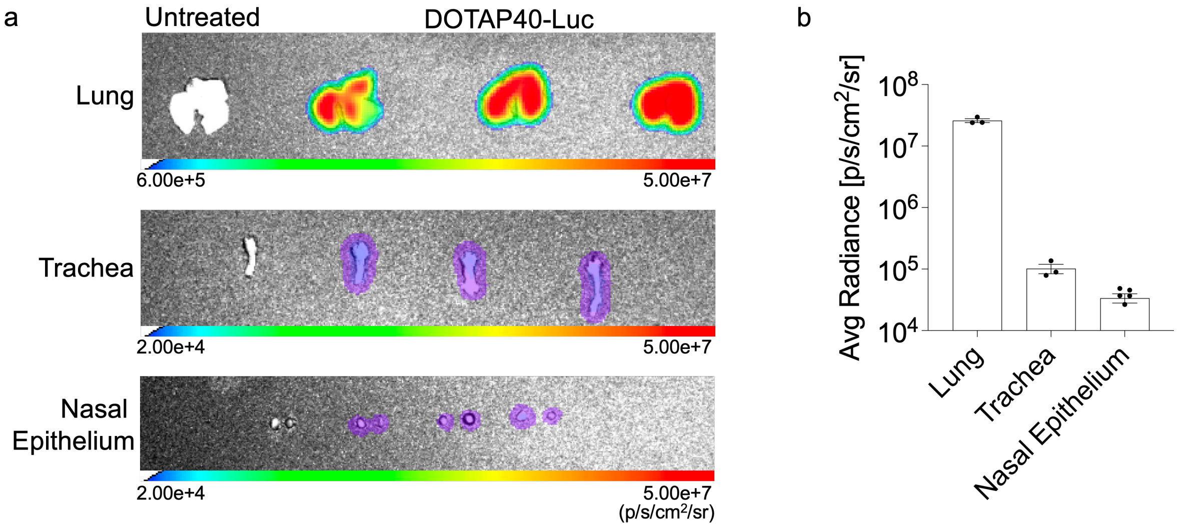

Supplementary Fig. 5 | DOTAP40 LNPs enabled functional mRNA delivery to airway tissues (lung, trachea, and nasal epithelium). C57BL/6 mice were treated with DOTAP40-Luc (Luc mRNA, 0.1 mg kg^{-1}, i.v., 20/1 (total lipid/total RNA)). *Ex vivo* airway tissue imaging (a) and quantification of luciferase expression (b) was performed 6 hours after the intravenous injection.

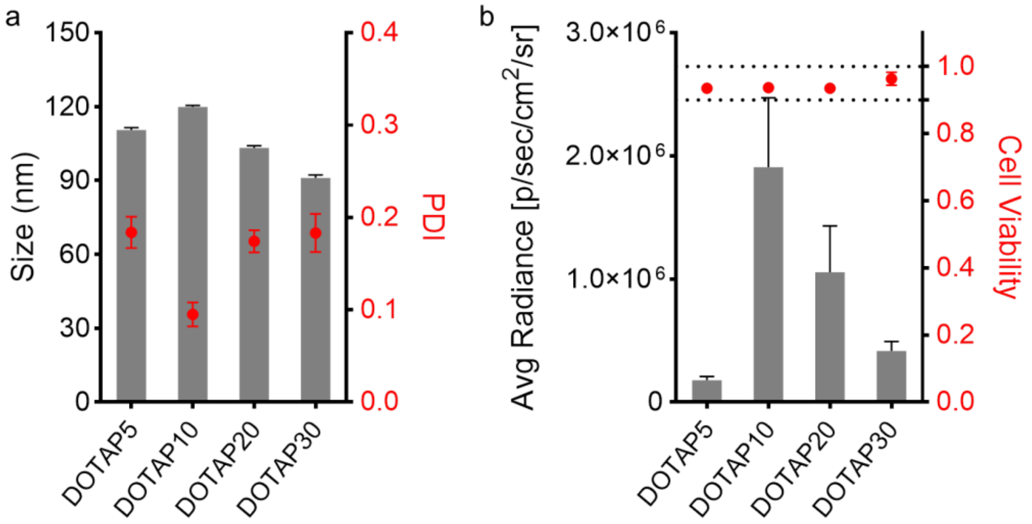

Supplementary Fig. 6 | DOTAP10 delivering Luc mRNA induced the highest luminescence intensity in HeLa cells. a, The size and polymer dispersity index (PDI) of a series of mDLNP-2 LNPs with 5%-30% DOTAP were measured by Dynamic Light Scattering (DLS). b, DOTAP10 LNPs efficiently delivered Luc mRNA to Hela cells and led to the highest luciferase expression after treatment of 24 hours with no obvious toxicity (20ng per well of Luc mRNA).

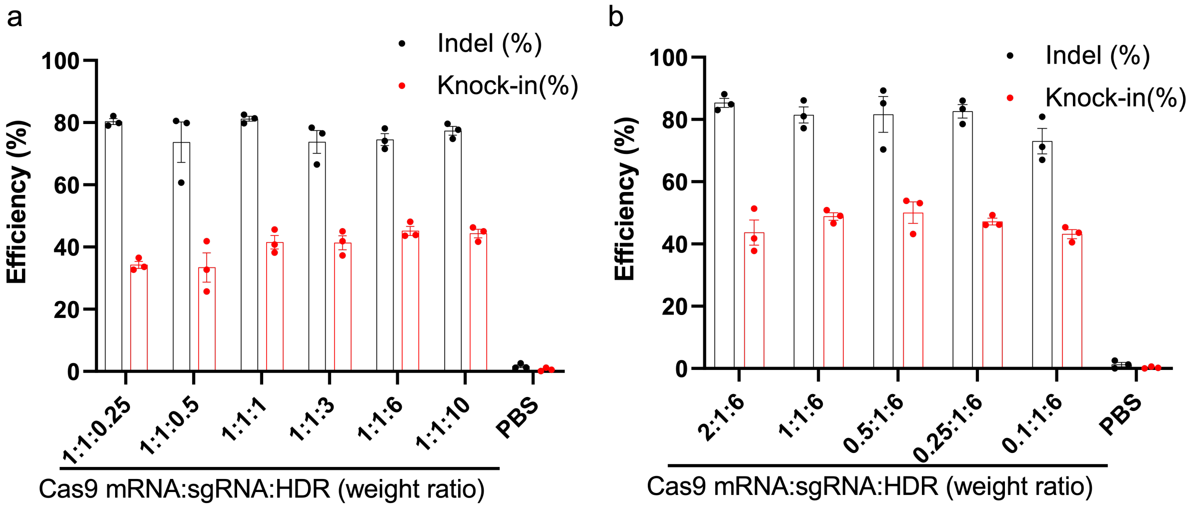

c

Cas9 mRNA/sgRNA/HDR (weight ratio)	Molar Ratio		
	Cas9 mRNA	sgRNA	HDR
1:1:0.25	1	45	10
1:1:0.5	1	45	19
1:1:1	1	45	38
1:1:3	1	45	114
1:1:6	1	45	229
1:1:10	1	45	382
2:1:6	1	23	114
0.5:1:6	1	91	458
0.25:1:6	1	181	916
0.1:1:6	1	453	2289

Supplementary Fig. 7 | DOTAP10 LNPs, encapsulating Cas9 mRNA, sgRNA and ssDNA HDR template at 0.5:1:6 (weight ratio) showed the highest HDR correction efficiency in HEK293 cells with Y66H GFP mutation. a, The Indel % and HDR % were analyzed using TIDER analysis with DNA sequencing data obtained after transfections with a series of DOTAP10 LNPs containing Cas9 mRNA:sgRNA weight ratio fixed at 1:1 (total NA at 0.8 ng μL^{-1}). b, The Indel % and HDR % were analyzed using TIDER analysis with DNA sequencing data obtained after transfections with a series of DOTAP10 LNPs containing sgRNA:HDR weight ratio fixed at 1:6 (total NA at 0.8 ng μL^{-1}). The optimal weight ratio among Cas9 mRNA, sgRNA and HDR was 0.5/1/6. Data are shown as mean±s.e.m. (n=3 biologically independent samples). c, The weight ratios and molar ratios of Cas9 mRNA/sgRNA/HDR used in the DOTAP10 LNP formulations.

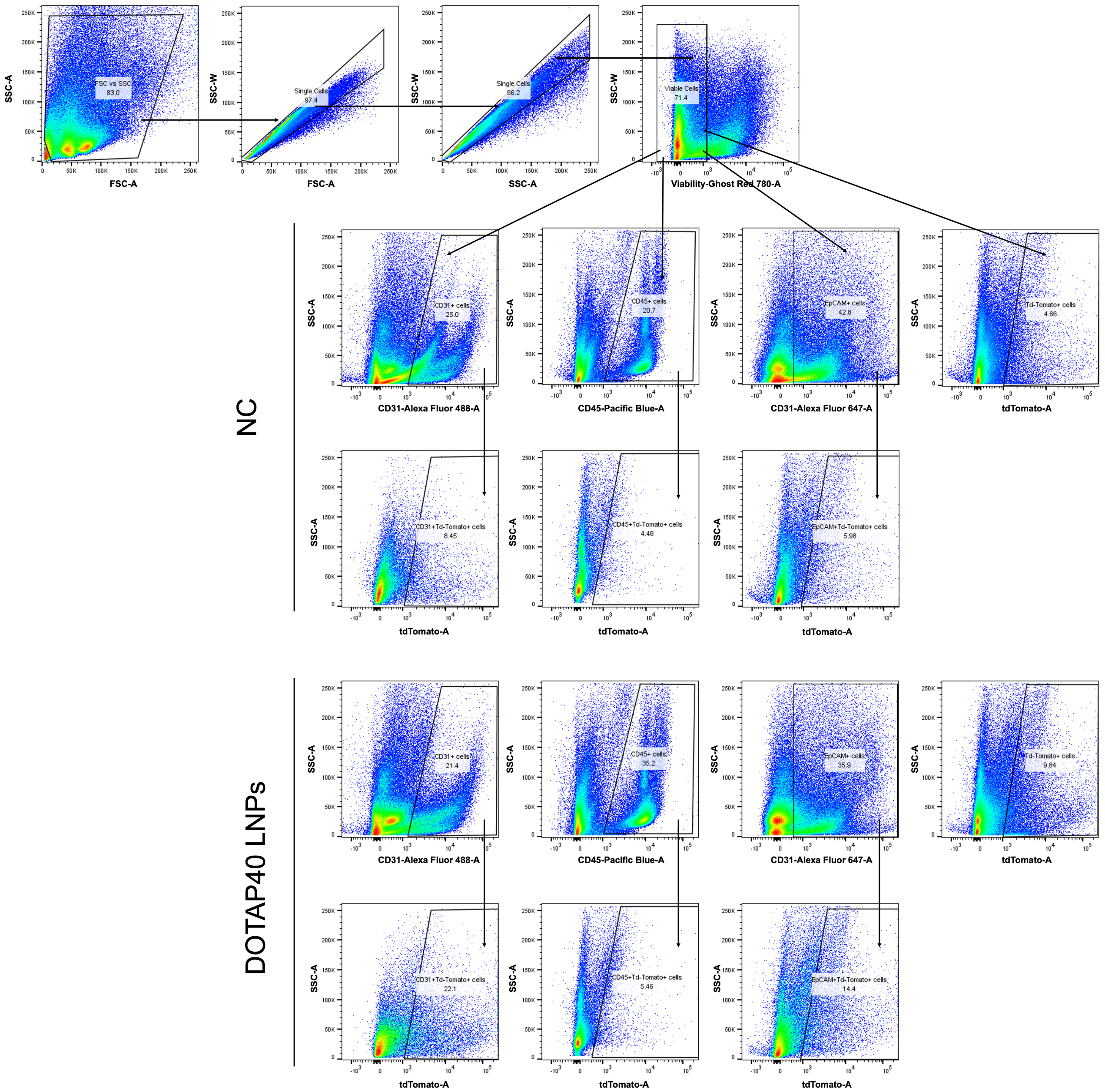

Supplementary Fig. 8 | Flow cytometry gating strategy for lung endothelial, epithelial, and immune cells. Single cells prepared from Ai14 mouse lungs were gated. Viable (Ghost Red negative) total lung cells, endothelial cells (CD31 positive), epithelial cells (EpCam positive), or immune cells (CD45 positive) expressing tdTomato fluorescence (tdTom positive) were analyzed by flow cytometry (n=3). Quantitative analysis was shown in Fig. 3g and 3h.

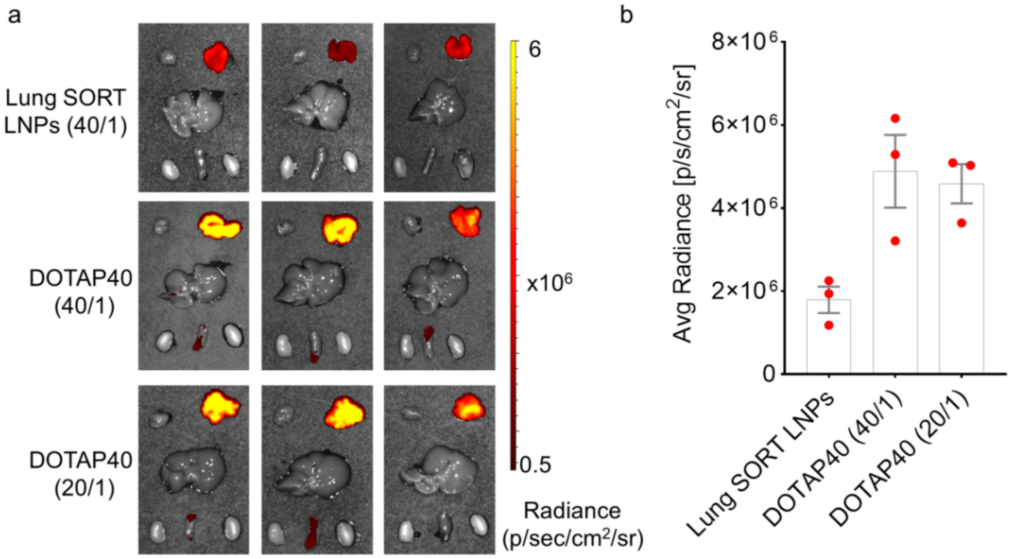

Supplementary Fig. 9 | The optimized DOTAP40 LNPs showed much higher luciferase expression in mouse lung, compared to Lung SORT LNPs reported. a, By adjusting the internal ratio among lipid components, the optimized DOTAP40 LNPs showed better delivery efficiency in mouse lungs than Lung SORT LNPs reported previously (0.1 mg kg^{-1} Luc mRNA). And we found that similar mRNA delivery efficiency was observed after decreasing weight ratio of total lipid/total NA from 40/1 to 20/1, detected using IVIS imaging system. b, Quantitative results after different treatment were measured from IVIS images obtained in a. Data are shown as mean±s.e.m. (n=3 biologically independent animals).

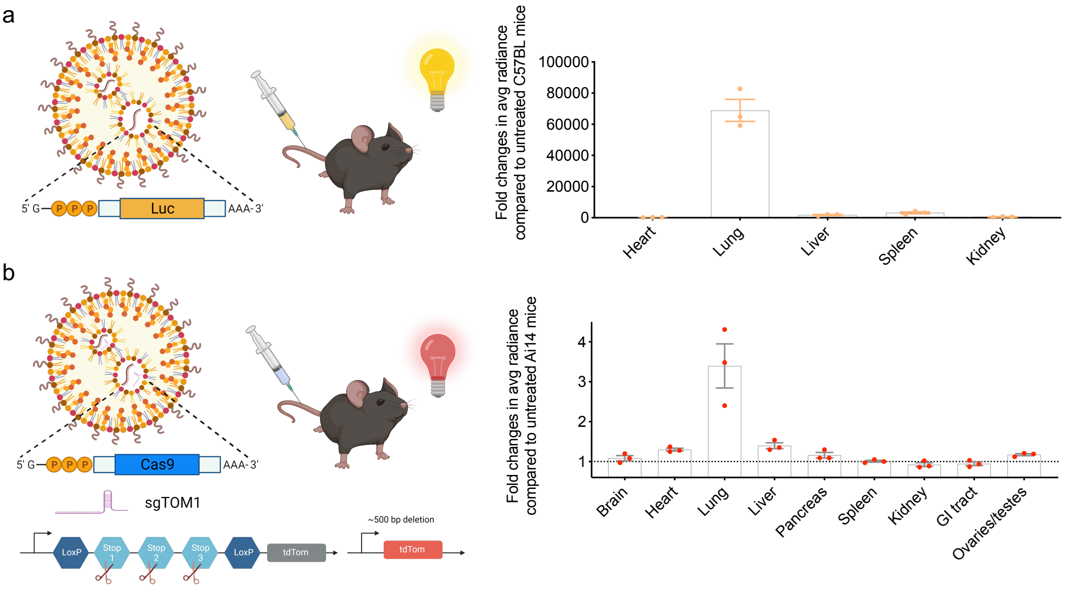

Supplementary Fig. 10 | Lung SORT DOTAP40 LNP facilities functional mRNA and gene editor delivery mainly in mouse lungs, with minimal off-organ delivery observed. a, Biodistribution of functional mRNA delivery after DOTAP40-Luc treatment. C57BL/6 mice were intravenously administrated with DOTAP40-Luc formulation (0.1 mg kg^{-1} Luc mRNA, 20/1 (total

lipid/total RNA)) once a week, three times in total. Mice were sacrificed 6 hours after the last injection and dissected to collect major organs (heart, lung, liver, spleen, and kidney). Biodistribution of functional luciferase mRNA was measured by *ex vivo* imaging using AMI imaging systems. Untreated C57BL/6 mice were used as control. Data are reported as fold changes in average radiance compared to untreated C57BL mice. b, Off-organ editing assessment after DOTAP40-NHEJ treatment. Ai14 tdTOM reporter mice were intravenously administrated with DOTAP40-NHEJ formulation (2 mg kg^{-1} Cas9 mRNA:sgTOM1=2:1, wt/wt, 20/1 (total lipid/total RNA)) once a week and three weeks in total. Mice were sacrificed one week after the last injection and dissected to collect organs (brain, heart, lung, liver, pancreas, spleen, kidney, GI tract and reproductive organs). Off-organ editing was assessed by measuring tdTOM fluorescence production after successful *in vivo* editing in reporter mice using AMI imaging systems. Untreated Ai14 mice were used as control. Data are reported as fold changes in average radiance compared to untreated Ai14 mouse. All data are shown as mean±s.e.m. (n=3 biologically independent samples).

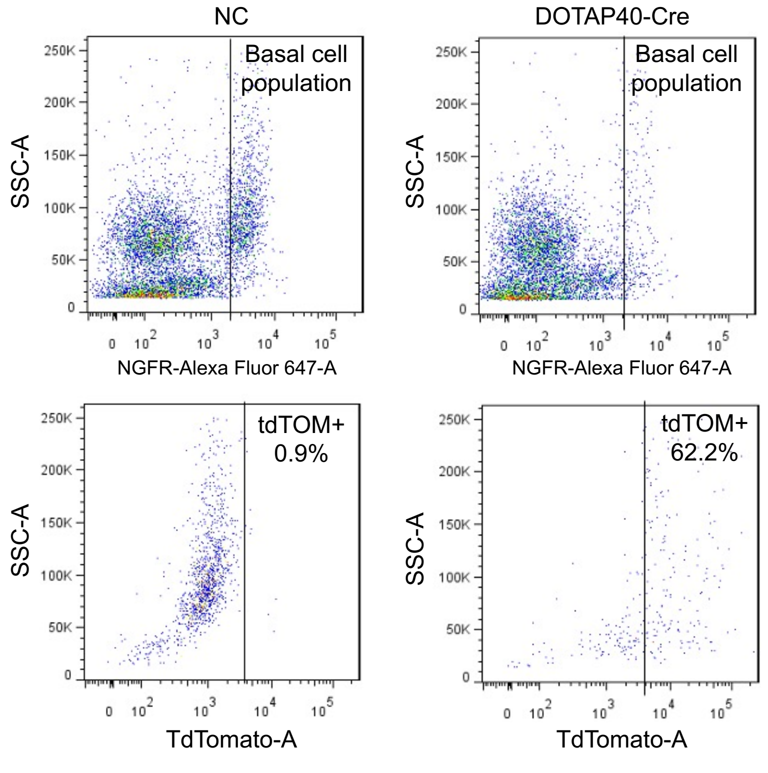

Supplementary Fig. 11 | Representative flow cytometry images showing that DOTAP40 LNPs efficiently delivered Cre mRNA to basal cells of tdTOM mouse lung and turned on tdTOM fluorescence. tdTOM mice were treated with DOTAP40 LNPs (20/1 (total lipid/total RNA)) encapsulating Cre mRNA twice by I.V. injection (2mg kg^{-1} Cre mRNA). Two days after the second injection, mouse lungs were collected and lung basal cell populations were sorted using Anti-p75 NGF receptor antibody and the tdTOM positive cells in the whole basal cell populations were analyzed using flow cytometry (n=4 biologically independent animals in DOTAP40-Cre group).

Quantitative analysis was shown in Fig. 4b. Nanoparticle only treatment group was used as negative control (NC).

a

Target	Gene	gRNA Sequence	PAM	MIT-Score	Off-target editing efficiency (%)
On Target	CFTR	GACAACACAGTTCTCTGAGA	AGG		
OT1	intergenic:-Mir568	G**C**C**C**ACACAGT**G**CTCTGAGA	GGG	1.38	0.04
OT2	intergenic:Plxnc1-Gm29684	**AGT**AACACA**T**TTCTCTGAGA	AGG	1.30	0.00
OT3	intron:Gxylt2	GACAACA**A**A**C**T**A**CTCTGAGA	GGG	1.10	0.04
OT4	intron:Xrcc4	**A**A**T**AA**G**A**G**AGTTCTCTGAGA	TGG	0.81	0.00
OT5	intergenic:Slc6a15-Gm8764/Gm6763/Gm4340/Gm21312/Gm21304/Gm21293/Gm20765	**AG**CA**TA**ACAGTTCTCTGAGA	GGG	0.79	0.04
OT6	intergenic:Syn2-Pparg	**C**A**CTG**GACAGTTCTCTGAGA	AGG	0.79	0.06

b

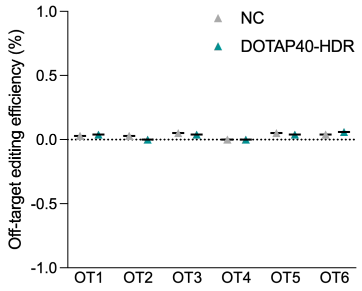

Supplementary Fig. 12 | No off-target editing was detected after treatment with DOTAP40-HDR nanoparticles. a. Sequence information of top six predicted off-target sites of sgG542X was listed in the table. b, The top 6 potential off-target sites were amplified by PCR and analyzed using NGS deep sequencing. NP only treatment group was used as negative control (NC).

a

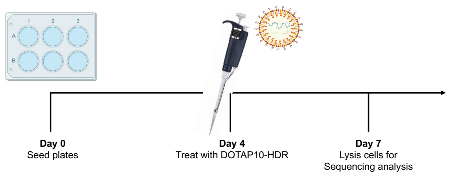

b

Name	Sequence of the HDR template (5' to 3')
HDR-NTS40	AGTTTTCCTGGATTATGCCTGGCACCATTAAAGAAAATATCATCTTTGGTGTTTCCTATGATGAATATAGATACAGAAGC
HDR-NTS60	AAGAATTTCATTCTGTTCTCAGTTTTCCTGGATTATGCCTGGCACCATTAAAGAAAATATCATCTTTGGTGTTTCCTATGATGAATATAGATACAGAAGCGTCATCAAAGCATGCCAACT
HDR-TS40	GCTTCTGTATCTATATTCATCATAGGAAACACCAAAGATGATATTTTCTTTAATGGTGCCAGGCATAATCCAGGAAAACT

c

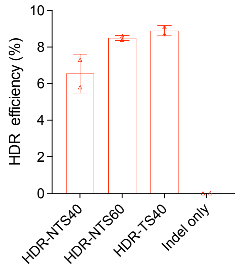

Supplementary Fig. 13 | HDR template optimization using P1 undifferentiated HBE culture with CFTR$^{F508del/F508del}$ mutation. a, Patient derived undifferentiated HBE cells with F508del mutation were treated with DOTAP10-HDR LNP for three days before cell collection for sequence analysis. b, A list of different HDR template targeting CF F508del mutation tested for HDR template optimization. c, HDR correction level analyzed by Sanger sequencing and TIDER analysis.

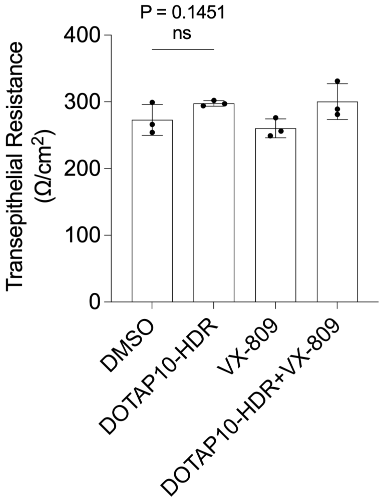

Supplementary Fig. 14 | Transepithelial resistance measurement using P3 fully differentiated HBE culture expressing mutant CFTR$^{F508del/F508del}$ after DOTAP10-HDR treatment. Data are shown as mean±s.e.m. (n=3). A two-tailed unpaired t-test were used to determine the significance of the comparisons of data (*P< 0.05; **P< 0.01; ***P< 0.001).

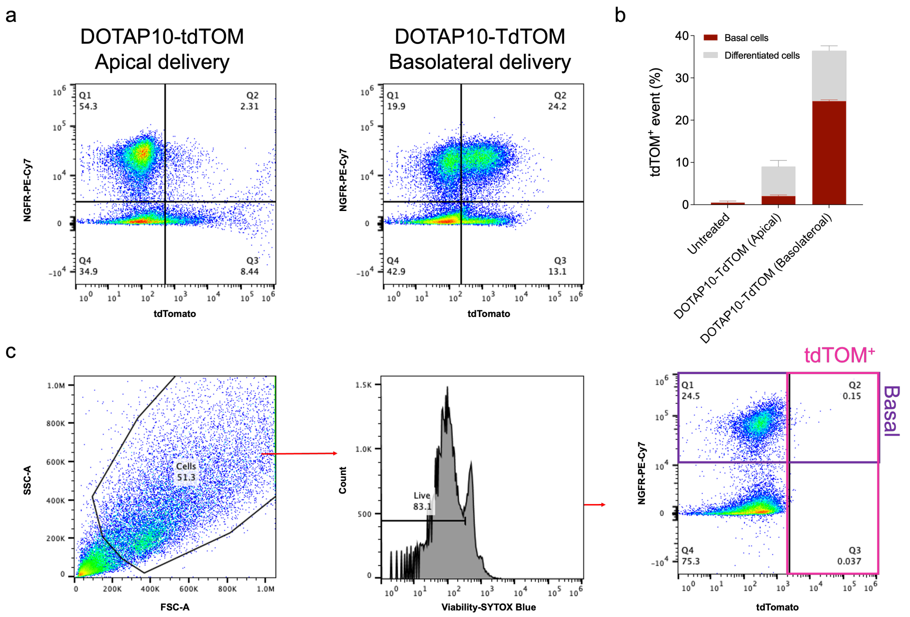

Supplementary Fig. 15 | Apical and basolateral delivery of DOTAP10-tdTOM in P3 fully differentiated HBE culture. a, Representative flow cytometry images (a) and quantitative analysis (b) of tdTOM fluorescent protein expressing (TdTOM^{+}) cells in HBE basal and differentiated cell populations. HBE cells were collected 24 hours after a single treatment of DOTAP10-tdTOM (12ug per well) from either apical side or basolateral side. Untreated HBE cells were used as control. c, Flow cytometry gating strategy for HBE basal cells. Single cells collected from each transwell were gated. Viable (SYTOX Blue) HBE basal cells (PE-Cy7 positive) expressing tdTOM fluorescence (tdTOM positive) were analyzed by flow cytometry. Data are shown as mean±s.e.m. (n=2).

Supplementary Tables

Supplementary Table 1. Characterization of DOTAP-HDR LNPs

**Formulation**	**Particle size (nm)**	**PDI**	**Zeta potential**	**% encapsulation**
DOTAP10-HDR	145.0 ± 0.8	0.12 ± 0.02	1.34 ± 2.20	72.3 ± 1.5
DOTAP40-HDR	137.0 ± 0.6	0.15 ± 0.01	7.57 ± 0.45	106.0 ± 1.1

Supplementary Table 2. Information of all sgRNA sequences used in this research.

Target	Target Sequences (5’ to 3’)	PAM (5’ to 3’)
sgBGFP	GCTGAAGCACTGCACGCCAT	GGG
sgG542X	GACAACACAGTTCTCTGAGA	AGG
sgF508del	ACCATTAAAGAAAATATCAT	TGG

Supplementary Table 3. Information of all ssDNA HDR template sequences used in this research.

Target	ssDNA HDR Template (5’ to 3’)
BGFP	GCCACCTACGGCAAGCTGACCCTGAAGTTCATCTGCACCACCGG<br>CAAGCTGCCCGTGCCCTGGCCCACCCTCGTGACCACCCTGACGTA<br>CGGCGTGCAGTGCTTCAGCCGCTACCCCGACCACATGA
G542X	TTTCAATTTTGGATTGTGCATGCTAAATTTTATTTCTGGTGTTATG<br>CTTTGGATAATAGGACATCACCAAGTTTGCAGAACAAGACAACA<br>CAGTTCTAGGAGAAGGTGGAGTCACACTGAGTGGAGGTCAGCGT<br>GCAAGGATTTCTTTAGCAAGGTAAATATTTAACTGTTGGTCTTGT<br>GAGCACTTGCTGTAAATACTA
F508del	AAGAATTTCATTCTGTTCTCAGTTTTCCTGGATTATGCCTGGCAC<br>CATTAAAGAAAATATCATCTTTGGTGTTTCCTATGATGAATATAG<br>ATACAGAAGCGTCATCAAAGCATGCCAACT

Supplementary Table 4. Information of primer sequences used for sanger sequencing.

Target	Forward Primers (5’ to 3’)	Reverse Primers (5’ to 3’)
BGFP	AAGCTTGGTACCGAGCTCG	TTGTGGCGGATCTTGAAGTTCAC
G542X	TGAATGGCACTTGAGTTTATATGAT<br>GGG	GAAATTCAAATCAGAGGCATCTGG<br>GGA
F508del	TGGGGGCAAGTGAATCCTGAGC	CATTGAGGACGTTTGTCTCACTAAT<br>GAGT

Supplementary Table 5. Information of primer sequences used for NGS on-target analysis (Illumina adapter sequences are underlined).

Target	Forward Primers (5' to 3')	Reverse Primers (5' to 3')
G542X	TCGTCGGCAGCGTCAGATGTGTATAAGAGACAGTCTGAAGAAAAATGTTCTTT	GTCTCGTGGGCTCGGAGATGTGTATAAGAGACAGCCATAGTATTTACAGCAAGT

Supplementary Table 6. Information of primer sequences used for NGS off-target analysis.

Target	Forward Primers (5' to 3')	Reverse Primers (5' to 3')
OT1	GTTTCCTCTGGTGAAACACTA	CAACTAAAGAATGACCATCCTAAC
OT2	GAGATTACTGACTCGTTGGAAT	AGAGAAACCCTTCGGTACA
OT3	TCAAGAATATGACTTCCTTCTATGG	GCAAGAGAGCCAAGGATTC
OT4	TTTCACTCTGCACTAGCAAA	TTGCTTCCATCACAGACTAAA
OT5	AGAATTACCAAGCTGCATCTAA	ATAGACCATAAGAGACCACAGA
OT6	TAATCTTGGAACTTGGCTTCTC	GTTGGATCACCAGCACAAA

Additional Supplementary References

1. Concordet, J.-P. & Haeussler, M. CRISPOR: intuitive guide selection for CRISPR/Cas9 genome editing experiments and screens. *Nucleic Acids Res.* **46**, W242-W245 (2018).
2. Doench, J.G. et al. Optimized sgRNA design to maximize activity and minimize off-target effects of CRISPR-Cas9. *Nat. Biotechnol.* **34**, 184-191 (2016).
3. Clement, K. et al. CRISPResso2 provides accurate and rapid genome editing sequence analysis. *Nat. Biotechnol.* **37**, 224-226 (2019).
