# Supplementary material for: Lung SORT LNPs enable precise homology-directed repair mediated CRISPR/Cas genome correction in cystic fibrosis models
Source: Nat Commun. 2023 Nov 11;14:7322. doi: 10.1038/s41467-023-42948-2 (PMC10640563; doi:10.1038/s41467-023-42948-2)
Supplement: Supplementary file 2 — Reporting Summary [file 41467_2023_42948_MOESM2_ESM.pdf]

nature portfolio

Corresponding author(s): Daniel J. Siegwart

Last updated by author(s): Oct 16, 2023

Reporting Summary

Nature Portfolio wishes to improve the reproducibility of the work that we publish. This form provides structure for consistency and transparency in reporting. For further information on Nature Portfolio policies, see our Editorial Policies and the Editorial Policy Checklist.

Statistics

For all statistical analyses, confirm that the following items are present in the figure legend, table legend, main text, or Methods section.

n/a	Confirmed	
☐	☒	The exact sample size (*n*) for each experimental group/condition, given as a discrete number and unit of measurement
☐	☒	A statement on whether measurements were taken from distinct samples or whether the same sample was measured repeatedly
☐	☒	The statistical test(s) used AND whether they are one- or two-sided<br>*Only common tests should be described solely by name; describe more complex techniques in the Methods section.*
☒	☐	A description of all covariates tested
☒	☐	A description of any assumptions or corrections, such as tests of normality and adjustment for multiple comparisons
☐	☒	A full description of the statistical parameters including central tendency (e.g. means) or other basic estimates (e.g. regression coefficient) AND variation (e.g. standard deviation) or associated estimates of uncertainty (e.g. confidence intervals)
☐	☒	For null hypothesis testing, the test statistic (e.g. *F*, *t*, *r*) with confidence intervals, effect sizes, degrees of freedom and *P* value noted<br>*Give P values as exact values whenever suitable.*
☒	☐	For Bayesian analysis, information on the choice of priors and Markov chain Monte Carlo settings
☒	☐	For hierarchical and complex designs, identification of the appropriate level for tests and full reporting of outcomes
☒	☐	Estimates of effect sizes (e.g. Cohen's *d*, Pearson's *r*), indicating how they were calculated

Our web collection on statistics for biologists contains articles on many of the points above.

Software and code

Policy information about availability of computer code

Data collection	Zetasizer software version 7.13 （Malvern Panalytical)<br>Living Image software version 4.3 (64-bit, Caliper Life Sciences)<br>Aura software v.4.0.7 (Spectral Instruments Imaging)<br>BD FACSDiva software version 8.0.1 (BD LSRFortessa)<br>BZ-X Viewer software version 1.0.0 (KEYENCE Corporation)<br>ZEN x64 software version 1.1.0 (Carl Zeiss Microscopy GmbH)<br>NDP.scan software version3.1.9 (NANOZOOMER, Hamamatsu)
Data analysis	GraphPad Prism 9.5.1 (GraphPad Software)<br>Aura software v.4.0.7 (Spectral Instruments Imaging)<br>Living Image software version 4.3 (Caliper Life Sciences)<br>TIDER Analysis tool (no version information available) (analyzing indels and HDR by decomposition)(http://shinyapps.datacurators.nl/tider/)<br>CRISPResso2 (analyzing NGS result to evaluate HDR efficiency)(https://crispresso.pinellolab.partners.org/)<br>FLOWJO software version 7.6 (FLOWJO)<br>BZ-X Analyzer software version 1.0.0 (Keyence Corporation)<br>ZEN 2010 software version 6.0.62 (Carl Zeiss MicroImaging GmbH)<br>NDP.view 2 software (version 2.7.25, Hamamatsu)

For manuscripts utilizing custom algorithms or software that are central to the research but not yet described in published literature, software must be made available to editors and reviewers. We strongly encourage code deposition in a community repository (e.g. GitHub). See the Nature Portfolio guidelines for submitting code & software for further information.

Data

Policy information about availability of data

All manuscripts must include a data availability statement. This statement should provide the following information, where applicable:

- Accession codes, unique identifiers, or web links for publicly available datasets
- A description of any restrictions on data availability
- For clinical datasets or third party data, please ensure that the statement adheres to our policy

All data needed to evaluate the conclusions in the paper are present in the main text, and supplementary material. The source data for the Figures along with the Supplementary Figures presented in this paper are available in the Source Data file. DNA sequencing files can be accessed at the National Center for Biotechnology Information Sequence Read Archive (NCBI SRA) with accession code PRJNA1012742. All other relevant data that support all findings within this paper are available from the corresponding author upon request.

Research involving human participants, their data, or biological material

Policy information about studies with human participants or human data. See also policy information about sex, gender (identity/presentation), and sexual orientation and race, ethnicity and racism.

Reporting on sex and gender	Not applicable.
Reporting on race, ethnicity, or other socially relevant groupings	Not applicable.
Population characteristics	Not applicable.
Recruitment	Not applicable.
Ethics oversight	Not applicable.

Note that full information on the approval of the study protocol must also be provided in the manuscript.

Field-specific reporting

Please select the one below that is the best fit for your research. If you are not sure, read the appropriate sections before making your selection.

[x] Life sciences [ ] Behavioural & social sciences [ ] Ecological, evolutionary & environmental sciences

For a reference copy of the document with all sections, see nature.com/documents/nr-reporting-summary-flat.pdf

Life sciences study design

All studies must disclose on these points even when the disclosure is negative.

Sample size	Sample sizes were determined using statistical power calculations. For various assays, we performed three to eight replicates to yield statistically significant differences.
Data exclusions	No data were excluded from the analyses.
Replication	Data reported were consistently replicated between three to eight times across multiple experiments with all replicates generating similar results.
Randomization	For in vivo animal experiments, mice were randomly allocated into each group. For in vitro and ex vivo cell-based experiments, cells were cultured under tightly controlled conditions.
Blinding	Due to the proof-of-concept nature of this study, true blinding of experiments was not possible. However, data collection and analyses for some experiments were conducted by separate individuals. In some cases, these collectors/analyzers were not aware which samples corresponded to which experimental groups at the time of data collection and analysis.

Reporting for specific materials, systems and methods

We require information from authors about some types of materials, experimental systems and methods used in many studies. Here, indicate whether each material, system or method listed is relevant to your study. If you are not sure if a list item applies to your research, read the appropriate section before selecting a response.

Materials & experimental systems

n/a	Involved in the study
☐	☒ Antibodies
☐	☒ Eukaryotic cell lines
☒	☐ Palaeontology and archaeology
☐	☒ Animals and other organisms
☒	☐ Clinical data
☒	☐ Dual use research of concern
☒	☐ Plants

Methods

n/a	Involved in the study
☒	☐ ChIP-seq
☐	☒ Flow cytometry
☒	☐ MRI-based neuroimaging

Antibodies

Antibodies used	Anti-p75 NGF Receptor antibody (ab8875) and Donkey Anti-Rabbit IgG H&L (Alexa Fluor® 647) (ab150075) was purchased from Abcam.<br>Anti-CFTR antibody (UNC 596) was purchased from University of North Carolina.<br>Anti-Actin antibody (MAB1501) was purchased from Millipore.<br>Anti-CD45 (Alexa fluor® 647)(#103124), Anti-EpCam (Alexa fluor® 647)(#324212) and Anti-CD31 (Pacific Blue)(#102422) were purchased from BioLegend.<br>IRDye 680RD Goat anti-Mouse IgG secondary antibody (926-68070) was purchased from LI-COR<br>PE-Cy7 anti-human CD271/NGFR (562122) was purchased from BD Biosciences
Validation	The antibody was validated internally validated using positive and negative control samples.<br>Validation/citation data are also available on the manufacturer's websites or CiteAb's website:<br>Anti-p75 NGF Receptor antibody (ab8875), https://www.citeab.com/antibodies/743864-ab8875-anti-p75-ngf-receptor-antibody?des=01b6c2b0e33ad8cf<br>Donkey Anti-Rabbit IgG H&L (Alexa Fluor® 647) (ab150075): https://www.abcam.com/products/secondary-antibodies/donkey-rabbit-igg-hl-alexa-fluor-647-ab150075.html?productWallTab=ShowAll<br>Anti-Actin antibody (MAB1501), https://www.sigmaaldrich.com/US/en/product/mm/mab1501<br>Anti-CFTR antibody (UNC 596), https://cftrantibodies.web.unc.edu/data-sheets/<br>Anti-CD45 (Alexa fluor 647)(#103124), https://www.biolegend.com/fr-fr/products/alexa-fluor-647-anti-mouse-cd45-antibody-3101<br>Anti-EpCam (Alexa fluor® 647)(#324212), https://www.biolegend.com/fr-ch/products/alexa-fluor-647-anti-human-cd326-epcam-antibody-3760<br>Anti-CD31 (Pacific Blue)(#102422), https://www.biolegend.com/en-us/products/pacific-blue-anti-mouse-cd31-antibody-6669?GroupID=BLG2420<br>IRDye 680RD Goat anti-Mouse IgG secondary antibody (926-68070), https://www.licor.com/bio/reagents/irdye-680rd-goat-anti-rabbit-igg-secondary-antibody<br>PE-Cy7 anti-human CD271/NGFR (562122), https://www.bdbiosciences.com/en-us/products/reagents/flow-cytometry-reagents/research-reagents/single-color-antibodies-ruo/pe-cy-7-mouse-anti-human-cd271.562122

Eukaryotic cell lines

Policy information about cell lines and Sex and Gender in Research

Cell line source(s)	Hela cells were originally obtained from ATCC.<br>BFP/GFP HEK293 cells were obtained from the laboratory of Professor Jacob Corn (ETH Zurich).<br>Primary HBE cells were collected from transplanted lungs from a healthy donor who has wild-type CFTRwt/wt and an individual with cystic fibrosis who was homozygous for CFTR F508del/F508del. Primary HBE cells were provided by the Cystic Fibrosis Foundation Therapeutic Lab.
Authentication	The cell lines were not further authenticated after receiving.
Mycoplasma contamination	The cell lines were not tested for mycoplasma contamination.
Commonly misidentified lines (See ICLAC register)	HeLa and HEK293 derivative cells are listed as a possible contaminating cell line according to ICLAC database.

Animals and other research organisms

Policy information about studies involving animals; ARRIVE guidelines recommended for reporting animal research, and Sex and Gender in Research

Laboratory animals	C57BL/6 mice were obtained from the UTSW Mouse Breeding Core Facility. B6.Cg-Gt(ROSA)26Sortm9(CAGtdTomato)Hze/J mice (also known as Ai9 or Ai9(RCL-tdT) mice) and B6.Cg-Gt(ROSA)26Sortm14(CAG-tdTomato)Hze/J mice (aslo known as Ai14 or Ai14(RCL-tdT)-D mice) were obtained from The Jackson Laboratory (007909)(007914) and bred to maintain in the UTSW Animal Facility. Mice bearing with homozygous G542X mutation were created by breeding heterozygous males and females by Cystic Fibrosis Mouse Models Core at Case Western Reserve University. Mice were housed in a barrier facility with a 12 h light/dark cycle and maintained

on standard chow (2916 Teklad Global). The temperature range for the housing room is 22 ºC and the humidity range is 35%-60% (average is around 50%).

Wild animals	The study did not involve the use of wild animals.
Reporting on sex	Both male and female mice were used. The sex of the animals was not further considered because LNP-mediated nucleic acid delivery data did not show bias toward one gender in experiments.
Field-collected samples	The study did not involve samples collected from the field.
Ethics oversight	C57BL/6 and Ai9/Ai14 mice experiments were approved by the Institution Animal Care and Use Committees of The University of Texas Southwestern Medical Center and were consistent with local, state, and federal guidelines as applicable. G542X mice experiment were approved by The Institutional Animal Care and Use Committee of Case Western Reserve University and were consistent with local, state, and federal guidance.

Note that full information on the approval of the study protocol must also be provided in the manuscript.

Flow Cytometry

Plots

Confirm that:

☒ The axis labels state the marker and fluorochrome used (e.g. CD4-FITC).

☒ The axis scales are clearly visible. Include numbers along axes only for bottom left plot of group (a 'group' is an analysis of identical markers).

☒ All plots are contour plots with outliers or pseudocolor plots.

☒ A numerical value for number of cells or percentage (with statistics) is provided.

Methodology

Sample preparation	For lung basal cell editing experiment, Lung tissue was minced using A blade and transferred to an EP tube containing 250µL of 2×digestion medium (90 units µL-1 collagenase I, 50 units µL-1 DNase I and 60 units µL-1 hyaluronidase, 1% FBS) and homogenized with tissue grinder. Afterwards, tissue solution was transferred to a 15mL centrifuge tube containing 10mL of 2×digestion medium and incubated at 37 °C for 1 hour with shaking. Next, the lung solution was filtered using a 70-µm filter and washed once with 1× PBS. A cell pellet was obtained by centrifuging 35 for 10min at a speed of 500×g at 4 °C. The supernatant was removed, and the cell pellet was resuspended in 2mL of 1×red blood cell lysis buffer (BioLegend, 420301) and incubated on ice for 5min. After incubation, 4mL of cell staining buffer (BioLegend) was added to stop red blood cell lysis. The solution was then centrifuged again at 500×g for 10min to obtain cell pellet. The single cells were resuspended in cell staining buffer to make cell solution at density of 1-5×106 cells mL-1. 100uL of cell solution was 40 transferred to a new EP tube to incubate with Anti-p75 NGF receptor antibody (1:100 dilution) for 30mins on ice, to stain lung basal cells. Afterwards, the cells were washed with 1× PBS buffer twice and incubated with Donkey Anti-Rabbit IgG H&L (Alexa Fluor® 647) (1:2000) for 30mins on ice, protecting from light (total volume 100 µL). The stained cells were washed twice with 1mL of 1× PBS, then resuspended in 500 µL 1× PBS for flow cytometry analysis using a LSRForessa SORP (version 45 8.0.1, BD Biosciences) in the Moody Foundation Flow Cytometry Facility. Ghost Dye Red 780 (TonboBiosciences, 13-0865-T500) was used to discriminate live cells.<br>For Cas9-mediated gene editing in TdTOM reporter mice experiment, mouse lungs were resected; and the tissues were placed into ice cold PBS. The lung tissue was then cut into small pieces and transferred into a 50 mL tube containing 10mL of 1X lung digestion media 20 [RPMI dissociation medium (1:1 vol/vol) RPMI supplemented with 2% wt/vol BSA, 300 U/mL collagenase, 100 U/mL hyaluronidase]. The 50mL tube was then incubated at 37 oC for 1hr while shaking at 180 rpm. After incubation, the homogenized lung cell solution was pipetted up and down several times to remove cell clumps and finally filtered through a 70-micron cell strainer into a new 50 mL falcon tube. The filter was washed with 10mL wash buffer consisting of cold PBS and 2% fetal bovine serum (FBS). 25 The sample was then centrifuged at 1200 rpm for 5 minutes. The supernatant was removed, and the cell pellet was resuspended in 10ml of cold wash buffer. Next, the red blood cells were lysed by resuspending the cell pellet in 5mL of 1X RBC lysis buffer (BioLegend) at room temperature for 5 minutes. After 5 minutes, 10mL of wash media was added to the sample. The sample was centrifuged at 1200rpm for 5 minutes. Finally, the RBC free cell pellet was resuspended in 5mL of cell staining buffer (BioLegend) 30 and proceeded with antibody staining for flowcytometry. Single-cell suspensions obtained from the mouse lungs were pre-blocked with mouse Fc-receptor blocker (BioLegend) for 15 minutes. Subsequently cells were labeled with an Alexa fluor 488-conjugated anti mouse CD31, Pacific, blue-conjugated anti mouse CD45 and Alexa fluor 647-conjugated anti mouse EpCAM antibodies (all from BioLegend) by incubating 100µL of cell suspension 35 with antibodies for 15 minutes on ice. Ghost dye red (BioLegend) was used to identify the dead cells. Next, the cell pellet was washed 3-times with cell staining buffer to remove excess antibodies. Finally, the cell pellet was resuspended in 500µL of cold cell staining buffer and kept on ice until analysis by flow cytometer.<br>For staining of HBE, FACS buffer was prepared at a final concentration of 5mM EDTA (Invitrogen, 15575-038), 10µM Y-27632 (STEMCELL, 72302), 10% FBS in 1X DPBS. Resuspended each cell pellet in 100µL of FACS buffer with PE-Cy7 anti-human CD271/NGFR (BD, 562122) at 1:100 dilution. Cells were incubated for 20 minutes in the dark at 4 °C. The stained cells were washed twice with 1mL 1X DPBS, then resuspended in 500µL FACS buffer with SYTOX Blue (ThermoFisher, S34857) at 1:1000 dilution to discriminate live cells. The HBE cells were then analyzed using ThermoFisher Attune CytPix machine (ThermoFisher) and FlowJo software (BD Life Sciences) to assess the DOTAP10-tdTOM uptake.
Instrument	BD FACSDiva software version 8.0.1 (BD LSRFortessa)
Software	FLOWJO software version 7.6 (FLOWJO)

Cell population abundance	Mouse lung cells were sorted based on cell surface marker NGFR expression (Alexa Fluor® 647)/EpCam (Alexa fluor 647)/ CD45 (Pacific blue)/ CD31 (Alexa fluor 488) followed by tdTomato tdTomato fluorescent protein expression using fluorescence-activated cell sorting (FACS) analysis. Purity of post-sort population was determined by FACS for Alexa Fluor® 647/Pacific, blue/Alexa fluor 488, then tdTomato.<br>Human HBE cells were sorted based on cell surface marker NGFR expression (PE-Cy7) followed by tdTomato tdTomato fluorescent protein expression using fluorescence-activated cell sorting (FACS) analysis. Purity of post-sort population was determined by FACS for PE-Cy7, then tdTomato.
Gating strategy	FSC/SSC and viability gating using Ghost Red Dye were applied for the flow cytometry experiment. Gating based on tdtomato positivity was used to detect of in vivo edited (tdTOM+) cells.

☒ Tick this box to confirm that a figure exemplifying the gating strategy is provided in the Supplementary Information.
